# Investigating the human spirit and spirituality in pediatric patients with kidney disease

**DOI:** 10.3389/fped.2023.1104628

**Published:** 2023-02-23

**Authors:** Robert Woroniecki, Michael L. Moritz

**Affiliations:** ^1^ Stony Brook Children’s, Stony Brook University, Stony Brook, NY, United States; ^2^ UPMC Children’s Hospital of Pittsburgh, University of Pittsburgh School of Medicine, Pittsburgh, PA, United States

**Keywords:** resilience, spirit, spirituality, social determinants of health, quality of life, religious beliefs, hope

## Abstract

Human spirit is an integral part of the medicinal art and science trifecta: body-mind-spirit, and it is contained in the World Health Organization definition of health. Human spirit is defined as our purpose in life, relationships with all living creatures or “Higher Power”, and in general our place on planet Earth. Spirituality is a required part of patient care according to Joint Commission on Accreditation of Health Care Organizations. There is an abundant medical literature that documents discrepancies in the results between studies and populations, and points to the importance of cultural, ethnic, spiritual or religious differences. Validated questionnaires used in research for last several decades demonstrated an association of spirituality with clinical outcomes, coping, and quality of life in different adult chronic diseases. There are also validated scales to measure hope in children based on the premise that children are goal directed and that their goal-related thoughts can be understood, yet their purposefulness, meaning of life and spirit in pediatric nephrology remains mostly unexamined. Although pediatric nephrology has made significant advances in molecular techniques, artificial intelligence, machine learning, and started to address more broad social issues such as racism, health equity, diversity of our work force, etc, it lacks both systematic ways of studying and philosophical approach to fostering human spirit. This mini review examines the place and knowledge gaps in human spirit and spirituality in pediatric nephrology. We review the concept of the human spirit and medical literature pertaining to its role in pediatric nephrology.

## Introduction

Kidney disease is fortunately not a common factor contributing to pediatric mortality. The primary causes of death in US American children, adolescent and young adults are fire arms, motor vehicle accidents, neoplasms, suffocation/drowning, drugs, or congenital anomalies (1). Over last few decades, pediatric nephrology has advanced with discoveries of several genetic, epigenetic and molecular mechanisms behind nephron injury and hypertension. Pediatric Nephrologists began recognizing the importance of other external factors that determine “renal health”, such as prematurity, race/ethnicity, socioeconomic factors, poverty, food insecurity, housing, immigration, physical activity, obesity, gender preference, and mental illness. An area that has not been well evaluated is the impact of human spirit in children with kidney disease.

## Human spirit

**Figure 1:**
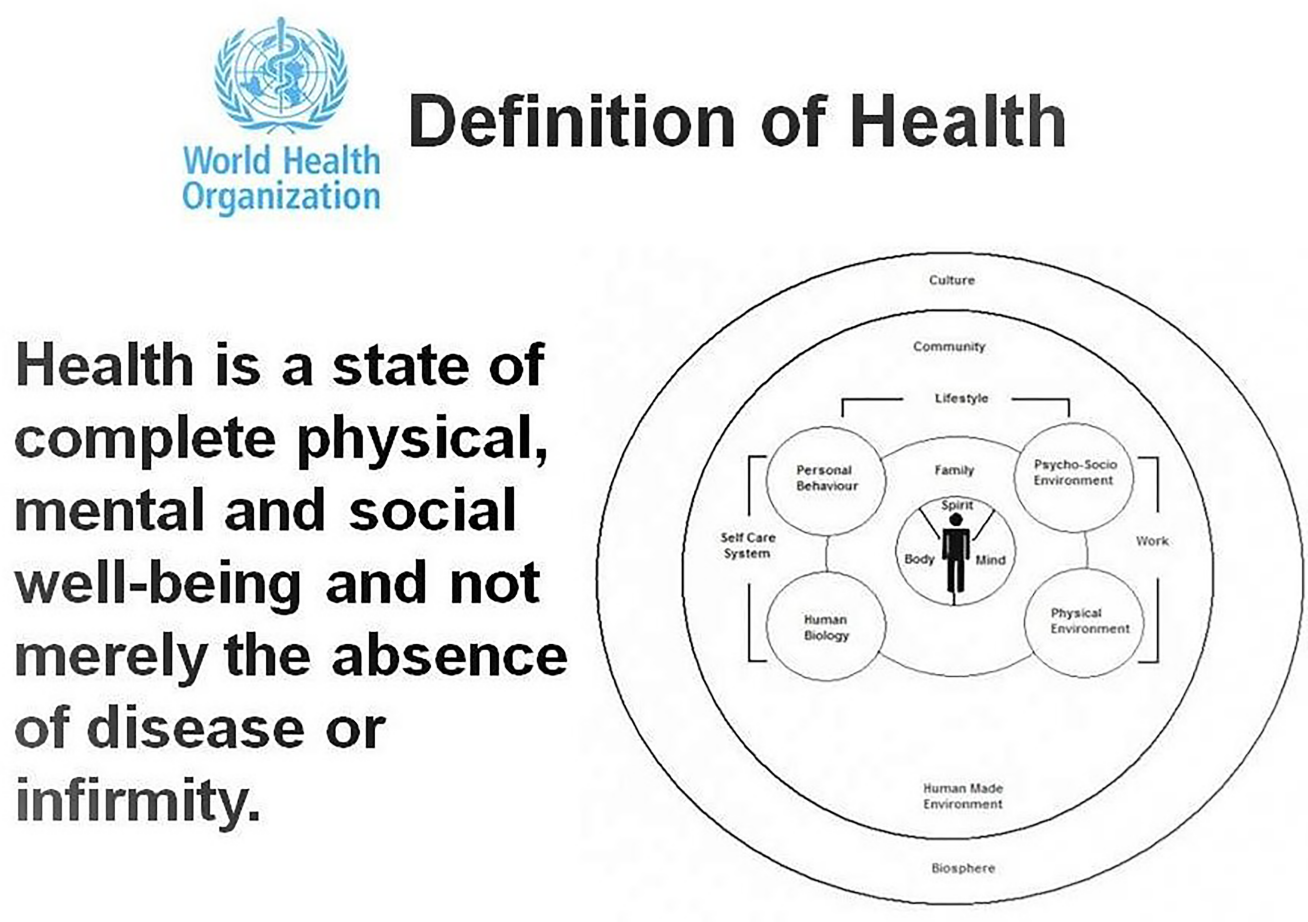
WHO definition of health. https://www.publichealth.com.ng/wp-content/uploads/2019/08/who-definition-of-health-1.jpg.

Spirit is a part of the medicinal art and science trifecta: body-mind-spirit. The World Health Organization (WHO) puts spirit at co-existence with body and mind in its definition of health (Figure 1) (2). Spirituality is also an integral part of patient care according to Joint Commission on Accreditation of Health Care Organizations, and specific instructions dealing with it were issued in 2005 and have remained since (3). Spirit has been an essential part of philosophical science throughout history, embracing the study of nature, principles of reality, knowledge, and values based on logic and reasoning (4).

## Spirit definition

The spirit and soul are not synonymous. They have had distinctive names in ancient cultures: in Hebrew they were called ruach and nephesh, which means “wind” and “breath” in Persian: ahu and urvan, in Buddhist and Hindu: monuhmed and gyan, and in Greek: pneuma and psyche (5). Spirit (“wind”) is the one that connects us to our external life, its purpose, relationship with other humans, animals, nature, or God. It is not our environment but rather a link “air” between the environment and our body and mind. Soul (“breath, psyche, mind”) connects us to the internal life, or our senses, emotions, desires, affections, and appetites, aches, body illness, etc. In Latin, the word spirit is related to the vital process of exchanging air, i.e., spirare means to breathe (6). Indigenous people belief that all nature-made (but not man-made) objects, for example, stone, lake, mountain, have their natural spirits (life purpose) (7). Since ancient times, scientists and philosophers like Aristotle recognized that the basic meaning of life refers to the ability of humans to formulate and implement purposes (8). Purposefulness is at the root of our species dominance allowing us to conquer the Earth, or travel to the Moon. Purposefulness is also one of humanities great shortcomings, allowing us to wage wars, eradicate other species, or other peoples (9). The role of human will was examined by Arthur Schopenhauer who proclaimed; “Man's world is based on man's will and is his idea of the world.” (10). Schopenhauer refers to will as the inner essence of everything: a blind, unconscious, aimless striving devoid of knowledge, outside of space and time, and free of all multiplicity. In his essay on the “free will”, he connects the will and self-consciousness (11). Albert Einstein summarized his philosophy as “a man can do as he will, but not will as he will” (12), and stressed importance of imagination over knowledge in creating solutions and human progress (13). If patients or parents cannot imagine solutions to problems life brings, they will not solve them.

## Role of spirit in medical arts

Our current US Healthy People 2030 government programs (14) focus on eliminating health disparities, creating equitable opportunities for people to live healthy lives, to advance health equity, increase health literacy, and address social determinants of health (SDOH). However, it should be noted that SDOH, are not equal to the human spirit, as they do not take into consideration the purpose of individual life. SDOH deal with conditions in the environments where people live, work, develop or age, and those factors are much easier to describe and quantify than individual human spirit formation. Patient economic stability, access to health, transportation, education, job, salary, exposure to violence, racism, etc, although important for health outcomes do not take into consideration individual spirit. SDOH do not define human spirit; do not seek its origin and the ways to foster it. It does not address basic need of human connections with Mother Nature, with human mother, does not specifically support fathers and inter-human relationships. Some peoples with similarly low salaries and similar exposure to stress, discrimination, etc., fare much better than others in terms of their mental and physical health.

One may hypothesize that perseverance of human spirit has a profound role in health outcomes. Those individuals who can “nourish” their spirit, find support, ways to effectively deal with, communicate, reach out, find friends, or simply escape stressful conditions may have better outcomes than those who cannot see or find solution, support, or cannot escape, and then sustain further damage to their spirit. Work of Victor Frankl, a survivor of Nazi concentration camp, revealed that the will to find meaning is the primary motivation in life. Human will, also termed “purpose in life” is associated with favorable health and survival outcomes including protective physiological and cognitive health benefits (15). In fact, logotherapy developed by Frankl (16) is a therapeutic approach focused on the future and on our ability to endure hardship and suffering through a search for purpose.

In adult studies, having purpose in life resulted in 17% less risk for all-cause mortality as well as cardiovascular events, including myocardial infarction, cardiac death, and stroke even after adjustment for sociodemographic, health, and functional status covariates (17).

Sense of purpose has been shown to be a critical factor in human functioning, disability and health. Human spirit could be affected by illness or injury of body or soul. Chronic disease can irreversibly alter purpose of life and human relationships, but human spirit can also affect body response to illness, injury or hardship (18). Higher purpose in life scores in adults predicted better health as measured by biomarkers 10 years later (19). It has been associated with fewer depressive symptoms in elderly (20) and with greater overall life satisfaction (21). The mechanisms underlying those findings include reduced levels of pro-inflammatory cytokines and inflammatory responses to stress (22). Psychological health is an important component of successful aging (23), with children having similar health and aging patterns to those found in their parents (24).

There is a substantial familial component to delayed onset of cardiovascular disease, hypertension, diabetes, and overall lower mortality (25, 26). Recent studies in children linked disorganized parenting and chaotic family life with latter in life cardio-vascular disease (27). We know based on epigenetic experiments that depending on the external experiences one has or does not have; some genes might be, or never be turned on (28). Maternal interpersonal problems are associated with disordered attachment and future relationship issues of the offspring (29). The direct connection of kidney disease with childhood adverse experiences (CAEs) or parental poverty is still lacking.

It is known that both under-nutrition and obesity is associated with childhood HTN, and that both of those conditions are associated with poverty, mental illness, poor relationships (30–33). There is a better mechanistic understanding on how microbiome affects hypertension (34), than how human spirit, social network, or family unit, presence of father and love of mother influence blood pressure regulation or renal outcomes. There are no observational or experimental studies in our field examining those factors.

Purpose in life has been associated with increased likelihood of seeking preventative health measures (35) and avoidance of risk taking behaviors (36).

On the personal and anecdotal level; when we talk to our patients about their complains, their medications, and tell them that most doctors address body and/or mind, and live out spirt, we cannot help but notice their facial and body expressions change, eyes become curious, and the dialog of what is really important to them develops. Patients also frequently thank us after, and say that nobody before talked to them about spirit, or that they “needed it”. Our trainees have a similar reaction.

Studies in pediatric nephrology of how human spirit and spirituality affects day-to-day life of children with chronic kidney disease or hypertension are lacking.

**Spirituality**, defined as a life oriented toward the spirit has close connection with religion and religious beliefs in kidney disease (37, 38). However, it should be noted that spirituality is distinct from religion understood as institutionally sanctioned beliefs and practices of a defined faith group (39). During evolution, human brain evolved and was able to imagine/meet God and through him/her/them define the individual life purpose, form community and common goals, and bear sacrifices as well as delay gratification. Judaism, the oldest monotheistic religion that has influenced the Christianity, Islam, and in general Western civilization, and addresses the ethical aspects of kidney disease treatment, transplantation, dialysis and end-of life issues (40, 41). Under Jewish written (Torah) and oral (Talmud) laws, with their origin of over 3 thousand years old, a physician is obligated to treat patients, not just to save a life but to restore health as well. According to those ancient laws, physicians have the obligation to prolong life even if a cure is not available, unless patient's suffering outweighs the benefit of life prolongation, and physicians are not allowed to “push away” one life for another (42).

Irrespective of the type of religion, philosophical pastoral care acts as a safeguarding of existence that helps patients to understand life better and to find a viable path in that moment when a person is at a loss (43). That is a way for hope, to find, even to invent, the connections that are so sustainable that it is possible to live in their network and to draw new strength from them, to wish and have confidence, or trust for a particular outcome. As with purposefulness, hope could also have drawbacks and unintended consequences when it becomes “false”, as mirage, and preventative of realistic choices (44).

In adult subjects on dialysis, the existential domain of spirituality had a greater impact on quality of life compared with measures of religiosity (37). However, in the field of pediatric nephrology the role of religion or spirituality or hope has not been well documented.

## Spirit measurements

Study and science of the soul/psyche (mind) is scientifically advanced. Soul (mind/psyche) and especially its physical location (brain) can be imaged, probed, stimulated, cut, and examined. Psychiatric disease has its classification and treatment (45). However, the study of the spirit without its physical location is much more difficult to accomplish. Yet, we aim to treat the patient as an individual or as a “whole” person as our field developed a quest for “personalized medicine” (46).

Validated spirituality questionnaires have been used in research for last several decades (47), and purpose of life can be measured in adults using standardized and validated tools (48, 49). Pediatric kidney disease literature focuses on measurements of quality of life (QOL) and does not measure human spirit or spirituality (50). In adults on dialysis, the spirituality measured by validated ESRD Spiritual Beliefs Scale (51) was clinically relevant and had an impact on health related QoL (37).

Although infants do not display characteristics purposeful action toward goals, as it requires learning to identify oneself (52), self-recognition is clearly in place by 12–18 months (53). Young children and adolescent are capable of learning complex abstract and scientific concepts (54, 55). In fact, there are validated scales to measure hope in children based on the premise that children are goal directed and that their goal-related thoughts can be understood according to two components: agency and pathways (56).

As of November 21, 2022 at 11:11AM PubMed search, not limited to English literature, with terms (https://pubmed.ncbi.nlm.nih.gov/?term=spiritual+health%2C+spiritual+well-being+and+children+and+kidney) showed 17 results; Table 1., and review of the subject showed no focus on the spirit/spirituality in children but rather on quality of life, experiences, SDOH, palliative care and religion.

**Table 1:** November 21, 2022 at 11:11AM pubMed search not limited to English literature with terms (https://pubmed.ncbi.nlm.nih.gov/?term=spiritual+health%2C+spiritual+well-being+and+children+and+kidney).

PMID	Title	Citation	First Author
31789430	Psychosocial interventions for preventing and treating depression in dialysis patients	Cochrane Database Syst Rev. 2019 Dec 2;12 (12):CD004542. doi: 10.1002/14651858.CD004542.pub3.	Natale P
29327140	Children's experiences of congenital heart disease: a systematic review of qualitative studies	Eur J Pediatr. 2018 Mar;177 (3):319-336. doi: 10.1007/s00431-017-3081-y. Epub 2018 Jan 11.	Chong LSH
33704475	Experiences of Latinx Individuals Hospitalized for COVID-19: A Qualitative Study	JAMA Netw Open. 2021 Mar 1;4 (3):e210684. doi: 10.1001/jamanetworkopen.2021.0684.	Cervantes L
27409075	Proceedings of the 3rd IPLeiria's International Health Congress: Leiria, Portugal. 6-7 May 2016	BMC Health Serv Res. 2016 Jul 6;16 Suppl 3 (Suppl 3):200. doi: 10.1186/s12913-016-1423-5.	Tomás CC
33729648	Stakeholder perspectives on the implementation and impact of Indigenous health interventions: A systematic review of qualitative studies	Health Expect. 2021 Jun;24 (3):731-743. doi: 10.1111/hex.13230. Epub 2021 Mar 17.	Chando S
26973911	Chronic Kidney Disease, Spirituality and Religiosity: A Systematic Overview with the List of Eligible Studies	Health Psychol Res. 2013 Aug 7;1 (2):e26. doi: 10.4081/hpr.2013.e26. eCollection 2013 Apr 18.	Bragazzi NL
29482461	Mothers’ Experience of Post-Traumatic Growth in Pediatric Kidney Transplantation	J Soc Work End Life Palliat Care. 2018 Jan-Mar;14 (1):110-123. doi: 10.1080/15524256.2018.1437587. Epub 2018 Feb 26.	Mantulak A
28971449	Clinical, Social and Demographics Factors Associated with Spiritual Wellbeing in End Stage Renal Disease	Adv Exp Med Biol. 2017;987:77-88. doi: 10.1007/978-3-319-57379-3_8.	Fradelos EC
23432815	Spiritual coping, religiosity and quality of life: a study on Muslim patients undergoing haemodialysis	Nephrology (Carlton). 2013 Apr;18(4):269-75. doi: 10.1111/nep.12041.	Saffari M
35807208	Palliative Care for Patients with Kidney Disease	J Clin Med. 2022 Jul 5;11 (13):3923. doi: 10.3390/jcm11133923.	Lanini I
33238900	How do parents deal with their children's chronic kidney disease? A qualitative study for identifying factors related to parent's adaptation	BMC Nephrol. 2020 Nov 25;21 (1):509. doi: 10.1186/s12882-020-02170-4.	Khorsandi F
28104711	Māori patients’ experiences and perspectives of chronic kidney disease: a New Zealand qualitative interview study	BMJ Open. 2017 Jan 19;7 (1):e013829. doi: 10.1136/bmjopen-2016-013829.	Walker RC
30525769	A longitudinal cohort study of symptoms and other concerns among Nigerian people with stages 3-5 chronic kidney diseases: study protocol	Ann Palliat Med. 2019 Apr;8 (2):190-198. doi: 10.21037/apm.2018.10.03. Epub 2018 Oct 17.	Olagunju AT
26082658	Ramadan fasting and chronic kidney disease: does estimated glomerular filtration rate change after and before Ramadan? Insights from a mini meta-analysis	Int J Nephrol Renovasc Dis. 2015 Jun 1;8:53-7. doi: 10.2147/IJNRD.S61718. eCollection 2015.	Bragazzi NL
25374827	Quality of life in end stage renal disease patients	World J Nephrol. 2014 Nov 6;3 (4):308-16. doi: 10.5527/wjn.v3.i4.308.	Joshi VD
24411716	Thematic synthesis of qualitative studies on patient and caregiver perspectives on end-of-life care in CKD	Am J Kidney Dis. 2014 Jun;63 (6):913-27. doi: 10.1053/j.ajkd.2013.11.017. Epub 2014 Jan 7.	Tong A

A recent systematic review of 8,946 articles regarding spirituality and health in adults (57) concluded that the role of spirituality in serious illness and health has not been systematically assessed. This is despite the fact that over 80 medical schools are teaching spirituality and compassionate care in medicine (58).

## Conclusions

WHO and JCO recommends addressing human spirit and spirituality in human health and disease Literature on applying this to children with kidney disease is lacking. Although our knowledge has advanced considerably in the field of human body and mind, and social determinants of health, and health equity take top priority in research government programs, human spirit remains under investigated, underappreciated, despite considerable evidence of its role in health outcomes. This mini-review is to raise awareness of the importance of the human spirit and to promote research on human spirit to public in benefit of us all.
